# Verruciform Xanthomas in the Setting of COVID-19: A Case Series and Review of Other Conditions Associated With This Benign Cutaneous Neoplasm

**DOI:** 10.7759/cureus.31849

**Published:** 2022-11-24

**Authors:** Osaigbokan P Aihie, Martin J Azzam, Adeeb Haroon, Kara Braudis

**Affiliations:** 1 Dermatology, University of Missouri, Columbia, USA; 2 Dermatology, Oregon Health & Science University, Portland, USA

**Keywords:** cell, foam, macrophage, infection, covid-19, neoplasm, benign, xanthoma, verruciform

## Abstract

Verruciform xanthoma is a rare benign neoplasm that predominantly affects the oral mucosa but can also affect cutaneous sites on the face, trunk, extremities, and genitalia. It is usually identified in isolation; however, there are several known associations with other conditions. Coronavirus disease 2019 (COVID-19) is a disease caused by severe acute respiratory syndrome coronavirus 2 (SARS-CoV-2), the coronavirus that emerged in December 2019 and caused a worldwide pandemic. It primarily manifests as a respiratory illness although various associations and sequelae of COVID-19 are still being elucidated. The clinical and pathologic presentations of two cases of Verruciform xanthoma associated with documented COVID-19 infection at our institution after the start of lockdowns during the COVID-19 pandemic in 2020-2021 are described. In addition, we reviewed the literature for other infectious and non-infectious diseases associated with Verruciform xanthomas to see if there is any basis for a potential link between this rare benign neoplasm and novel viral infection.

## Introduction

Verruciform xanthoma (VX) is a rare benign neoplasm, which predominantly affects the oral mucosa. The entity was first described by Shafer in 1971 and subsequently confirmed by additional reports [1]. Even rarer, cutaneous VX was first reported by Santa Cruz and Martin in 1979 as affecting the genitalia [2]. Subsequent reports confirmed the majority of cutaneous VXs affect the genitalia, but lesions on the face, trunk, and extremities have also been reported [3-5]. Clinically, the lesions appear as a singular well-demarcated papule or plaque, which exhibits papillary, verrucous, or “cauliflower-like” morphology [3,4]. Histologically, the lesions are characterized by a parakeratotic and papillomatous epidermis with varying amounts of distinguishing neutrophils [3-6]. The base of the lesion typically exhibits band-like infiltrates of inflammatory cells [3-6]. However, the defining histologic feature is aggregates of foam cells in the papillary dermis [3-6].

Despite these findings, the pathogenesis of this condition remains unclear. Immunohistochemical studies have shown that the foam cells stain positive for CD68, suggesting they are of monocyte-macrophage origin [7]. These findings have led to speculation of an abnormal immunologic response as the main driving force [3,7]. Various other conditions have been reported in association with VX. These include relatively common diseases, such as lichen planus or lupus, to more rare conditions like graft-versus-host disease (GVHD) or congenital hemidysplasia with ichthyosiform erythroderma and limb defects (CHILD) syndrome [3]. Despite these associations, our literature review revealed no previous reports of VX in association with COVID-19. Herein, we described the clinicopathologic presentation of two unique cases of VX after a documented COVID-19 infection.

## Case presentation

We review two cases of documented COVID-19-positive patients who had verifiable COVID-19 infection prior to a diagnosis of VX at the University Dermatology Clinic in Columbia, Missouri. This study examined VX patients seen at our outpatient clinic over the two-year period from the start of pandemic lockdowns in the U.S.A. in March 2020 through December 31, 2021. Over this timeframe, there were a total of seven patients seen at our dermatology clinic with biopsy-proven VXs - a higher number than the preceding several years combined. This prompted an evaluation to identify if there was any correlation between these two entities or if this was merely a coincidence. Table 1 lists all seven patients and highlights a breakdown between them. Patients 4 and 5 are discussed in further detail given their dual COVID-19 positivity and biopsy-proven VXs. 

**Table 1:** Breakdown of biopsy-proven Verruciform xanthoma patients, 3/2020 – 12/2021 SCC: Squamous Cell Carcinoma, MIS: Melanoma In-Situ, BCC: Basal Cell Carcinoma, SCCis: Squamous Cell Carcinoma In-Situ

Patient	Age, Sex	Location of lesion	Notable skin conditions	Recurrence after excision	COVID-19 positive prior to diagnosis
1	72 F	Finger	Irritant hand dermatitis, shingles	Yes	No
2	72 M	Nose	N/A	No	No
3	41 F	Lip	N/A	No	No
4	86 M	Ear	SCC, stasis dermatitis	No	Yes
5	66 M	Scrotum	SCC, MIS	No	Yes
6	85 M	Hand	BCC, SCC, Melanoma	No	No
7	66 M	Scrotum	SCCis, BCC	No	No

Patient 4 was an 86-year-old male with a past dermatologic history significant for non-melanoma skin cancer, who was originally seen on 9/2/2021 for bilateral lower extremity eczematous dermatitis superimposed upon stasis dermatitis. Prior to this visit, he was diagnosed with COVID-19 on 8/6/2021. At the time of his dermatology appointment, the patient’s COVID symptoms had subsided, and it was recommended he be managed with compression stockings and triamcinolone 0.1% topical ointment for his cutaneous symptoms, with anticipated follow-up in one month.

**Figure 1:**
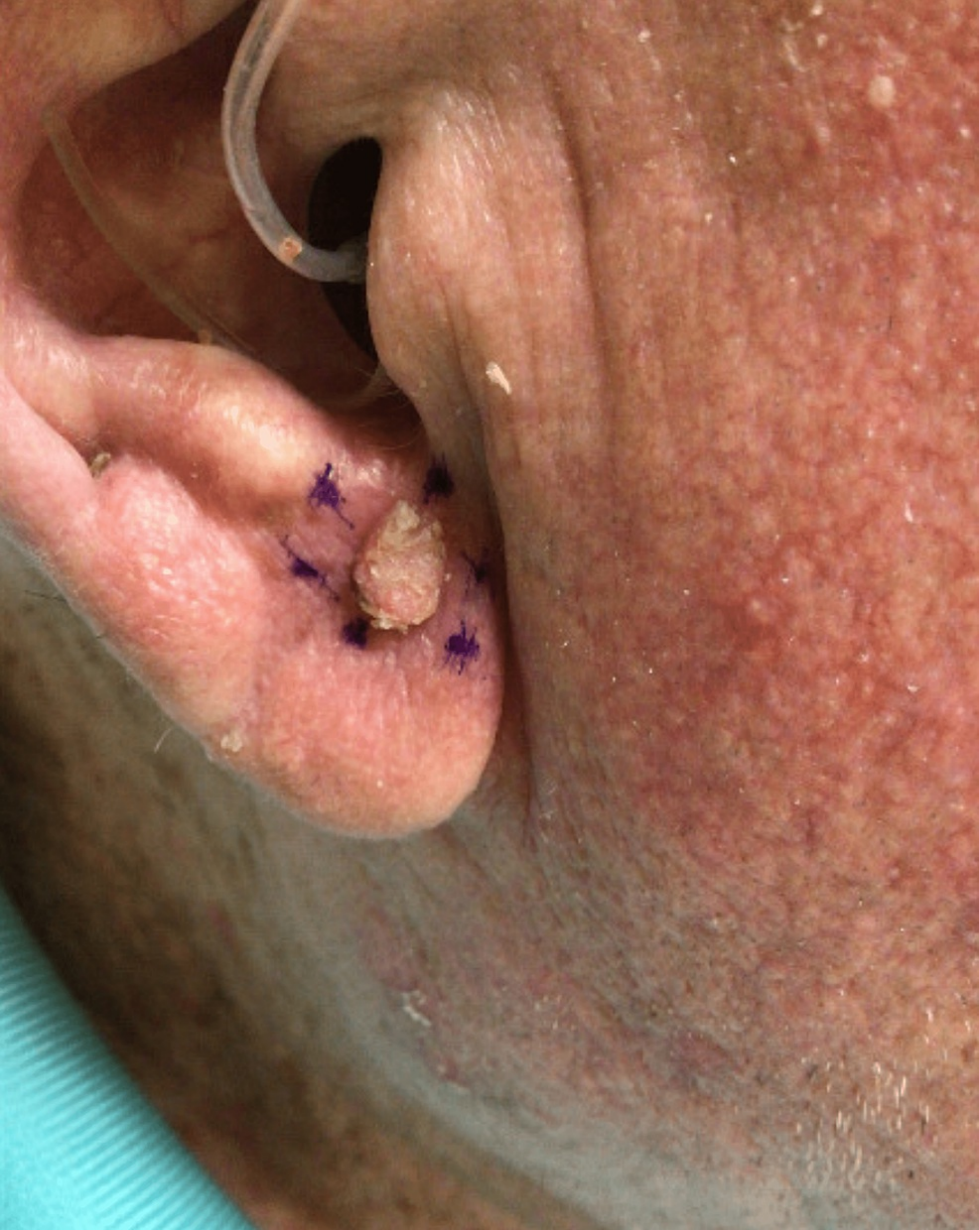
Clinical presentation of Verruciform xanthoma on the right ear lobule of an 86-year-old man The lesion, outlined in purple ink, consists of a 6 mm pedunculated hyperkeratotic papule on the right ear lobule.

**Figure 2:**
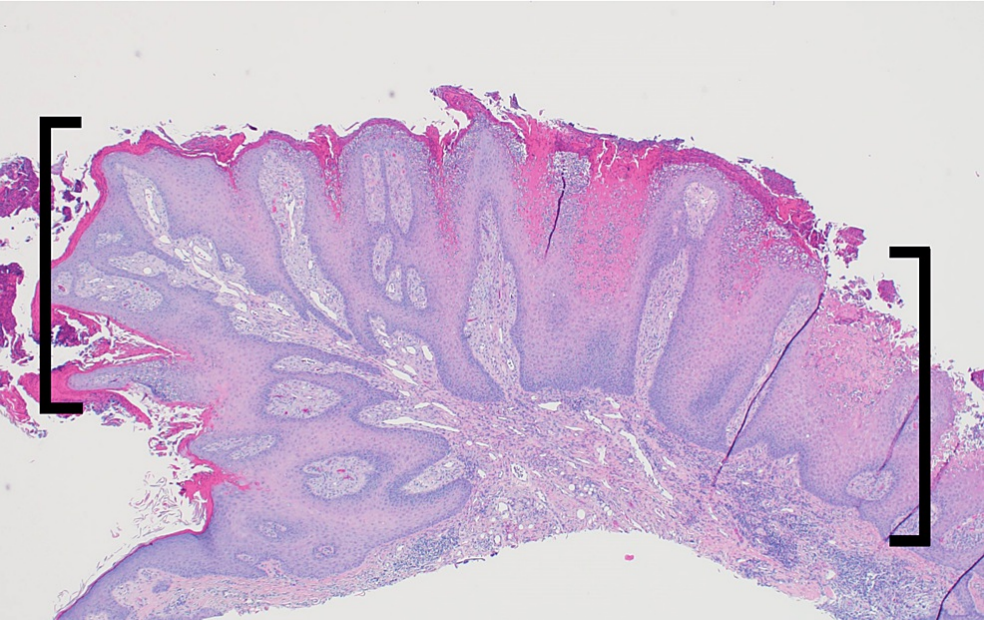
Pathology presentation of Verruciform xanthoma on the right ear lobule of an 86-year-old man Shave biopsy of the pedunculated hyperkeratotic papule demonstrates the characteristic features of a Verruciform xanthoma at low power, specifically the papillated epidermal hyperplasia (black brackets) (Hematoxylin and eosin: x4).

**Figure 3:**
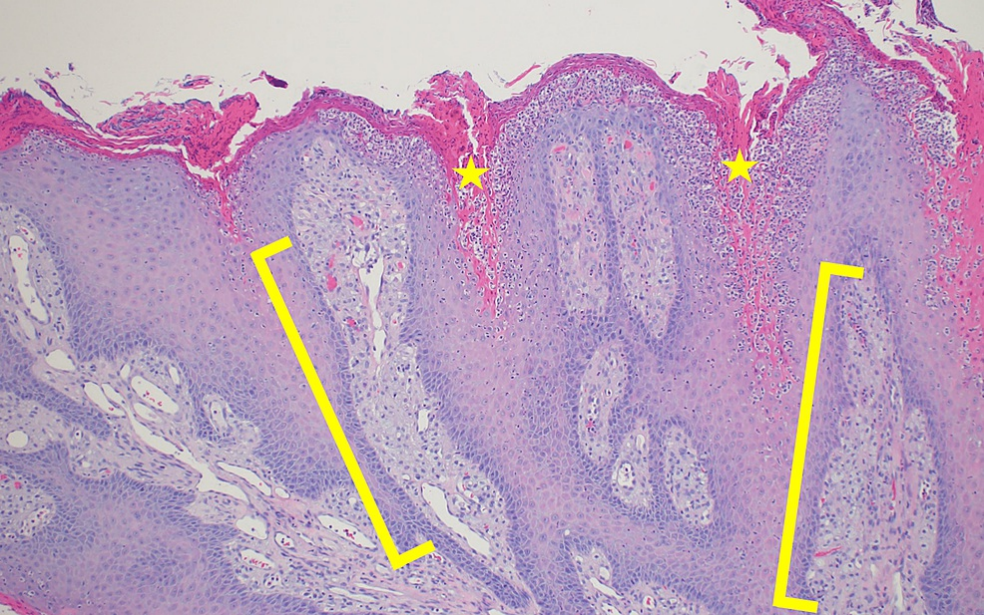
Pathology presentation of Verruciform xanthoma on the right ear lobule of an 86-year-old man Shave biopsy of the pedunculated hyperkeratotic papule demonstrates the characteristic features of a verruciform xanthoma at higher power, specifically the foamy macrophages (yellow brackets) and neutrophilic infiltrates (yellow stars) (hematoxylin and eosin: x10).

When the patient presented for his one-month follow-up visit on 10/4/2021, dermatitis on his bilateral lower extremities had significantly improved. However, he had two lesions, he was unaware of and that were concerning for cutaneous malignancies: a 6 mm pedunculated hyperkeratotic papule on the right ear lobule (Figure 1), and a 5 mm hyperkeratotic tender papule on the right dorsal middle finger. The lesion on the patient’s right dorsal middle finger returned as a squamous cell carcinoma and was treated at the time of biopsy via shave removal plus curettage and cryotherapy. While the lesion on the right ear lobule returned as a VX, with the biopsy showing papillated epidermal hyperplasia with foamy macrophages in widened dermal papillae and neutrophilic infiltrates (Figures 2, 3).

Neither lesion has resurfaced since 10/4/2021 and the patient has elected for the observation of both sites without further treatment. He returns to the clinic regularly every six months for a total body skin examination given his history of non-melanoma skin cancers.

Patient 5 was a 66-year-old male with a past dermatologic history significant for melanoma in situ (right cheek, status post excision in 2015) and squamous cell carcinoma (left forearm, status post excision in 2009), who was presenting for his annual skin check on 10/14/2021. Prior to this visit, he was diagnosed with COVID-19 on 12/12/2020, which ultimately resulted in his being admitted to the ICU and requiring the use of a ventilator for six days. However, the patient eventually made a full recovery and his COVID-19 symptoms had long subsided by the time of his dermatology appointment.

**Figure 4:**
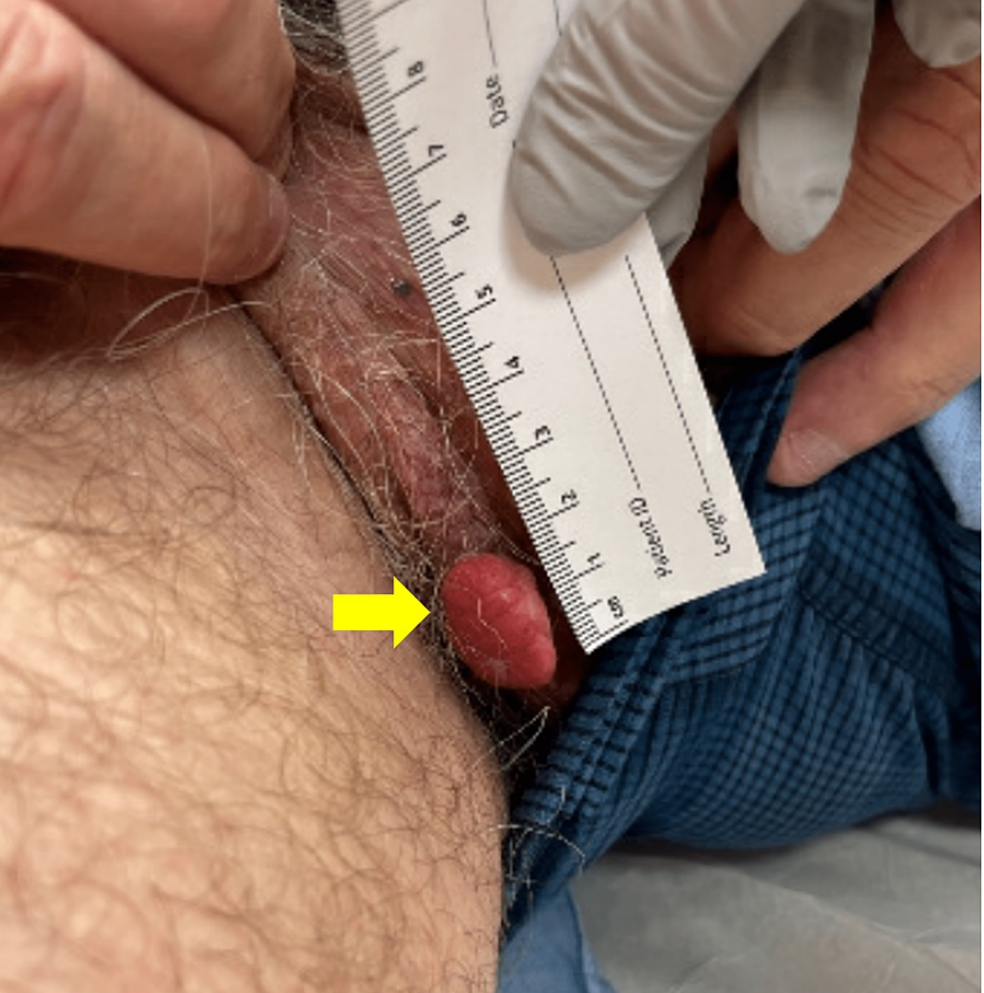
Clinical presentation of Verruciform xanthoma on the right scrotum of a 66-year-old man The lesion, being pointed to with the yellow arrow, consists of an erythematous pedunculated verrucous papule on the right scrotum.

His chief complaint at this visit was the desired removal of a 2.0 cm erythematous pedunculated verrucous papule on the right scrotum (Figure 4). The patient indicated that this lesion gradually appeared over the past several months and was bleeding, causing him a great deal of discomfort. A shave removal was performed. Dermatopathology of the lesion showed papillated epidermal hyperplasia with foamy macrophages in widened dermal papillae and neutrophilic infiltrate (as in Figures 2, 3), most consistent with a VX.

The patient has not had any recurrence of this lesion to date. He returns to the clinic annually for a total body skin examination given his past dermatologic history of melanoma in situ and squamous cell carcinoma.

## Discussion

Verruciform xanthomas (VX) are rare benign neoplasms that mainly affect the oral cavity. In the mouth, VXs are commonly found on the gingiva and the hard palate as raised or flat yellow-to-red verrucous lesions [8]. Other common oral sites include the lower and upper alveolar ridges, floor of the mouth, tongue, and buccal mucosa [9-14]. The oral lesions are usually solitary, slow-growing, and painless. They can range anywhere between 2 mm to 2 cm in diameter [15,16]. Oral VXs are more common in men than women and appear after the fifth decade of life [17,18]. There are, however, reports of lesions in individuals younger than 30 years and even as young as 14 years, suggesting that these lesions can present at any age [10,19].

VXs can also arise extra-orally, especially in anogenital regions, including the vulva, scrotum, penis, and anus [2,20-22]. In very rare occasions, VXs can arise in non-anogenital cutaneous regions like the ear, nose, hand, and neck [15,23-25]. As in the mouth, cutaneous VXs are small and appear papillomatous and reddish-to-yellow in color [26]. While most cases of cutaneous VXs are solitary, cases of multiple lesions have been reported, including concomitant involvement of both genital areas and oral mucosa [26]. When VX appears on the oral mucosa or skin, the lesions are frequently mistaken for other more common oral or cutaneous conditions like leukoplakia, verruca vulgaris, and verrucous carcinoma [15]. A biopsy is thus needed to confirm the diagnosis.

On histology, VX lesions show uniformly elongated papillae that project from the dermis with parakeratosis and, most importantly, foam cells [1]. These foam cells are mainly within the papillae and rarely extend into the layers beneath [1]. They are positive for CD68 and arise from a macrophage lineage [7,27]. Other inflammatory infiltrates comprised of plasma cells, lymphocytes, eosinophils, and most characteristically neutrophils surround the foam cells [8].

While the histologic and immunohistochemical features of VX have been well-delineated, the pathogenesis of this condition still remains unclear. Several hypotheses have focused on explaining the presence of lipids in the foam cells of VX. Zegarelli et al. proposed that the lipids are released from degenerating epithelial cells, with the subsequent arrival of macrophages to phagocytize the debris, thus leading to the formation of foam cells [28]. However, Nowparast et al. proposed that the macrophages may appear first, and then the epithelial cells secondarily cause a change in the metabolism and architecture of these cells, hence leading to foam cells [29]. Finally, Moshin et al. proposed yet another mechanism of pathogenesis similar to Zegarelli et al., suggesting that an unknown event initiates damage to keratinocytes leading to the production of specific cytokines. These cytokines then attract macrophages, which move into the area, phagocytize the defective keratinocytes, and transform into foam cells [4].

The presence of VXs mainly on the oral mucosa frequently in contact with food, suggests that inflammation may play a crucial role in this lesion’s pathogenesis [17]. An inflammatory etiology is further supported by the predominance of multiple inflammatory cell types within VX. Since VX has been found in conjunction with many other skin conditions, such as pemphigus vulgaris, epidermal nevus, and lupus erythematosus, it is also possible that the pathogenesis of this neoplasm may be somehow linked to other cutaneous disorders [30-32].

With regard to infectious diseases, VX has been linked to human papillomavirus (HPV). This is because both lesions appear similar clinically and histologically, as well as affect anogenital areas. While few studies have found HPV in VX lesions [33,34], many more have not [8,34-37]. Most of the studies that did not find HPV in VX lesions set out to look for a link between the two disease processes [3]. This suggests that reports of HPV in VX lesions are incidental [3]. Thus evidence for HPV causing or being linked to VX is weak. Blankenship et al. provided a summary table showing various skin diseases that have been associated with reports of VX between 1980 and 2012 [3]. Table 2 provides an updated comprehensive summary of skin conditions reported with VX over the past decade.

**Table 2:** Reported cases of Verruciform xanthoma associated with other skin conditions since 2012 GVHD: Graft-Versus-Host Disease, CHILD Syndrome: Congenital Hemidysplasia With Ichthyosiform Erythroderma and Limb Defects Syndrome, RDEB: Recessive Dystrophic Epidermolysis Bullosa, KID Syndrome: Keratitis-Ichthyosis-Deafness Syndrome

Author	Year	No. of Cases	Location	Associated Skin Condition
Wu et al. [37]	2006	1	Abdomen	Seborrheic keratosis
Sibaud et al. [38]	2006	1	Oral cavity	GVHD
Poulopoulos et al. [39]	2007	1	Oral cavity	Systemic lupus erythematosus
Ko et al. [40]	2008	1	Face, neck, trunk, and lower extremities	Linear epidermal nevus
Orpin et al. [41]	2008	1	Shoulder	Dystrophic epidermal bullosa
Anbinder et al. [42]	2010	1	Oral cavity	Neurofibromatosis and lichen planus
Kurban et al. [43]	2010	1	Foot	CHILD syndrome
Fedda et al. [44]	2011	1	Foot	CHILD syndrome
Farahani et al. [45]	2011	5	Oral cavity	Chronic GVHD
Fite et al. [5]	2011	10	Vulva	Lichen sclerosus
Xu et al. [46]	2013	1	Vulva and left lower limb	CHILD syndrome
Simon et al. [47]	2017	1	Leg	Leg ulcer
Keyal et al. [48]	2017	1	Scrotum	Neurofibroma
Theofilou et al. [49]	2018	1	Tongue	Oral lichen planus
Evan-Browning et al. [50]	2019	1	Back	RDEB
Evan-Browning et al. [50]	2019	1	Thigh and foot	KID syndrome
Stephens et al. [51]	2019	1	Thigh	RDEB
Jenkyn et al. [52]	2019	1	Oral cavity	GVHD
Chiang et al. [53]	2020	1	Knee	RDEB
Jiali and Jiang [54]	2021	1	Vulva	Epidermal nevus

Taking into consideration the link between VX and infectious disease, our findings seem to be in concordance with the literature. The present two case series reports were the only patients with both documented COVID-19 infection and VX out of the seven total VX patients seen in our clinic between the start of pandemic lockdowns in the U.S.A in March 2020 through December 2021. The fact that only 2/7 (29%) VX patients seen in our clinic had documented COVID-19 infection lends further credence to the weak association between this benign neoplasm and infectious disease.

Lastly, regarding treatment, VX usually requires surgical excision [55]. However, the lesion has been known to re-occur after resection [27,56]. The complete resolution has been reported on various occasions. Connolly et al. described the surgical excision of a lesion in the inguinal fold, which resurfaced but finally resolved with the application of a 10% povidone-iodine solution for six months [57]. Joo et al. reported complete resolution of a scrotal lesion after shave debulking and fractionated laser CO_2_ treatment with a wavelength of 10,600 nm [58]. In addition, Guo et al. reported complete resolution of VX of the labia minora with imiquimod 5% topical cream [59].

## Conclusions

Though several hypotheses exist to explain the pathogenesis of verruciform xanthoma, its etiology still remains unclear. Furthermore, though Verruciform xanthoma is associated with various other conditions, including infection in the form of HPV, this association is weak and thought to be incidental. In light of the literature review and the poor relationship between our patients with Verruciform xanthomas and documented COVID-19 infection, the authors of this paper concluded that there is likely no causal link between Verruciform xanthomas and COVID-19. Some limitations of this study include the potential of other case series' patients to have had undocumented COVID-19 infection, as well as the overall scarcity of this benign cutaneous neoplasm in the general population. The relative influx of patients with verruciform xanthomas during the pandemic was probably coincidental and due to a multitude of factors ranging from increased medical information consumption to personal awareness of overall well-being in this unusual time of heightened health anxiety.
